# Correlation between the numbers of rotation steps in the ATPase and proton-conducting domains of F- and V-ATPases

**DOI:** 10.1007/s12551-020-00668-7

**Published:** 2020-04-08

**Authors:** Hiroyuki Noji, Hiroshi Ueno, Ryohei Kobayashi

**Affiliations:** Applied Chemistry, Graduate School of Engineering, The University of Tokyo, Tokyo, 113-8656 Japan

## Abstract

This letter reports the correlation in the number of distinct rotation steps between the F_1_/V_1_ and F_o_/V_o_ domains that constitute common rotary F- and V-ATP synthases/ATPases. Recent single-molecule studies on the F_1_-ATPase revealed differences in the number of discrete steps in rotary catalysis between different organisms—6 steps per turn in bacterial types and mitochondrial F_1_ from yeast, and 9 steps in the mammalian mitochondrial F_1_ domains. The number of rotational steps that F_o_ domain makes is thought to correspond to that of proteolipid subunits within the rotating *c*-ring present in F_o_. Structural studies on F_o_ and in the whole ATP synthase complex have shown a large diversity in the number of proteolipid subunits. Interestingly, 6 steps in F_1_ are always paired with 10 steps in F_o_, whereas 9 steps in F_1_ are paired with 8 steps in F_o_. The correlation in the number of steps has also been revealed for two types of V-ATPases: one having 6 steps in V_1_ paired with 10 steps in V_o_, and the other one having 3 steps in V_1_ paired with 12 steps in V_o_. Although the abovementioned correlations await further confirmation, the results suggest a clear trend; ATPase motors with more steps have proton-conducting motors with less steps. In addition, ATPases with 6 steps are always paired with proton-conducting domains with 10 steps.

## ATP synthase

**Fig. 1:**
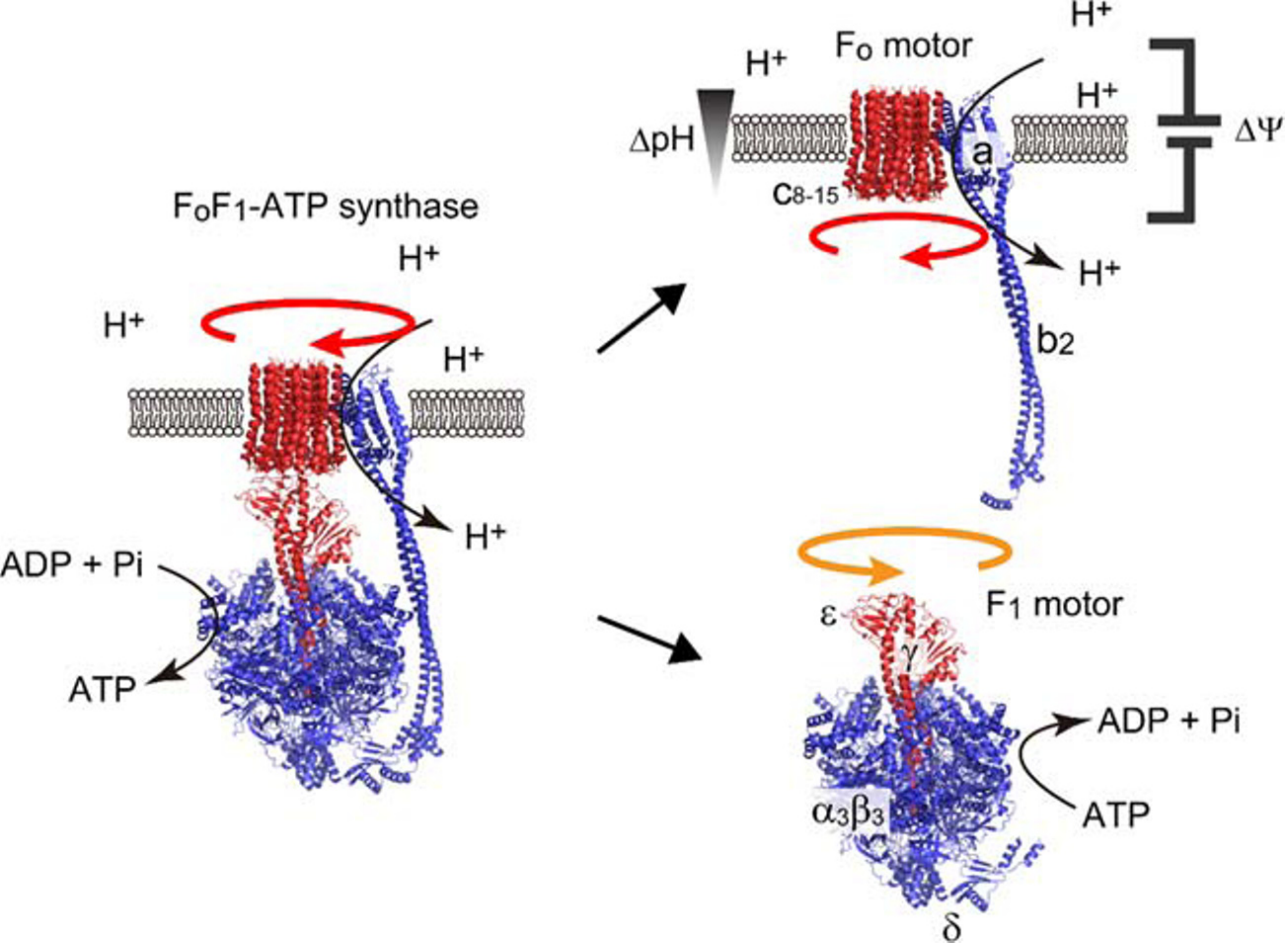
The two rotary motors of ATP synthase, F_1_ and F_o_. The subunit composition of F_1_ and F_o_ in bacterial types is α_3_β_3_γδε and *ab*_2_*c*_*n*_, respectively, where *n* varies among species. F_1_ rotates the rotary shaft, composed of the γ and ε subunits (red) against the α_3_β_3_ stator ring (blue). F_o_ rotates the oligomer ring of the *c*-subunits (red) against the *ab*_2_ stator complex (blue) during proton translocation across the membrane. In the whole ATP synthase complex, the rotor complexes F_1_ and F_o_ form the common rotary shaft (red) and stator complexes (blue), which are connected via the peripheral stalk formed by the *b*_2_ and δ subunits

The ATP synthase, also known as F_o_F_1_ ATPase or F-ATPase, mediates the energy interconversion between the proton motive force (*pmf*) across membranes and the free energy of ATP hydrolysis via a rotary catalysis mechanism (Abrahams et al. 1994; Yoshida et al. 2001; Noji et al. 2017). The ATP synthase is composed of two rotary motors, F_1_ and F_o_ (Fig. 1) (Junge et al. 1997). F_1_ is the catalytic core domain responsible for ATP synthesis, showing an active ATPase activity when isolated (Yasuda et al. 2001; Spetzler et al. 2006; Bilyard et al. 2012; McMillan et al. 2016). Upon ATP hydrolysis, F_1_ rotates the inner subunit (γε) against the catalytic stator ring (α_3_β_3_). F_o_ is the membrane-embedded domain and conducts proton translocation across the membrane. Upon proton translocation, F_o_ rotates the oligomeric ring formed by the proton-carrying *c*-subunits against the stator complex (ab_2_). In the whole ATP synthase complex, the rotor parts of F_1_ and F_o_ are bound together, forming the common rotary shaft (Junge et al. 1997; Oster and Wang 2000; Yasuda et al. 2001). The stator parts of F_1_ and F_o_ are connected via the peripheral stalk to transmit the torque without slippage. When *pmf* is sufficient, F_o_ generates a larger torque than F_1_, reversing the rotation of the rotor shaft in F_1_ to induce ATP synthesis. In contrast, when *pmf* is low, F_1_ reverses the rotation of the rotor ring in F_o_, forcing F_o_ to actively pump protons and generate *pmf*.

## Stepping rotation of F_1_

The minimum F_1_ complex as a rotary motor is the α_3_β_3_γ subcomplex, which rotates the rod-shaped γ subunit against the α_3_β_3_ stator ring in a counterclockwise direction when viewed from the F_o_ side. The catalytic reaction centers for ATP hydrolysis reside at the three pairs of α−β, with the main catalytic residues harbored in each of the β subunits (Weber and Senior 1997). The three β subunits conduct the catalytic reaction in a highly sequential manner, resulting in a sequential power-stroking conformational change that rotates the γ subunit unidirectionally.

As expected from the pseudo threefold symmetry of F_1_, the unitary rotational step is 120° rotation, coupled with a single turnover of ATP hydrolysis (Yasuda et al. 1998). The rotation dynamics of the γ subunit in F_1_ from thermophilic *Bacillus* PS3 (TF_1_) has been intensively characterized to establish a standard reaction scheme for bacterial F_1_ domains. TF_1_ makes 80° and 40° sub-steps in a single 120° rotation, which means that TF_1_ makes rotational steps intervened with 6 pauses per turn (Yasuda et al. 2001; Shimabukuro et al. 2003; Nishizaka et al. 2004; Adachi et al. 2007). Other F_1_ domains from bacteria and mitochondrial F_1_ from yeast (*y*MF_1_) were reported to make 6 pauses per turn (Steel et al. 2015). Thus, a 6-step rotation is widely conserved across microorganism species.

On the other hand, rotation assays in mammalian F_1_ domains have found an additional pause in 120° rotation, which translates into 9 steps per turn (e.g., three step rotations at 65°, 25°, and 30°) in human mitochondrial F_1_ (*h*MF_1_) (Suzuki et al. 2014). Similarly, bovine mitochondrial F_1_ (*b*MF_1_), the gold standard model for structural analysis of F_1_, was studied in the rotation assay and found to have an additional pause in 120° rotation (Kobayashi et al. 2020). However, the position in *b*MF_1_ is different from *h*MF_1_, making three step rotations of 10–20°, 60–70°, and 40°. These observations suggest that a 9-step rotation is conserved in mammalian mitochondrial F_1_ domains.

We should be able to progressively detect smaller sub-steps by improving the spatiotemporal imaging resolution and the data analysis methods. In fact, we analyzed the data of rotation trajectories with elaborated mathematical methods and found that TF_1_ makes an additional small step of 10° between the 80° and 40° sub-steps (Li et al. 2015). In this review, we aimed at a coarse-grained classification of the rotation behavior of F_1_. Therefore, we only considered the experimentally distinctive steps: the step size must be over 10°, and/or the intervening pause must be long enough to set the pace of the overall rotation rate under a certain condition, typically in the range of sub- or milliseconds.

## Stoichiometry of H^+^ per turn of F_o_

F_o_ is a membrane-embedded motor with the minimum subunit composition of *a*_1_*b*_2_*c*_n_. The stoichiometry (*n*) of the *c*-subunits varies from 8 to 15 among species (Meier et al. 2005; Pogoryelov et al. 2009; Watt et al. 2010; Saroussi et al. 2012; Preiss et al. 2014, 2015; Morales-Rios et al. 2015; Guo et al. 2019). The *c*-subunits form an oligomer ring that is rotated against the *ab*_2_ stator complex upon proton translocation across the membrane. According to the two half-channel model (Vik and Antonio 1994; Junge et al. 1997), which is well supported by the recent cryoEM studies, the *a*-subunit has two half-channels, one exposed on each side of the membrane (Allegretti et al. 2015). Each proton enters through one of the half-channels and is transferred to one of the *c*-subunits. After one turn of the *c*-ring against the *ab*_2_ stator, the proton is transferred to the other half-channel of the *a*-subunit facing the opposite side of the membrane. Thus, a proton is translocated by a *c*-subunit, and therefore, the total number of protons translocated per turn is determined by *n,* the number of *c*-subunits in the oligomer *c-*ring.

Currently, there are not enough reports on the stepping rotation of F_o_ to discuss the experimental data in a comprehensive manner. Our working assumption is that the number of steps in F_o_ is determined by *n*.

## Number of steps in F_1_ versus F_o_

**Fig. 2:**
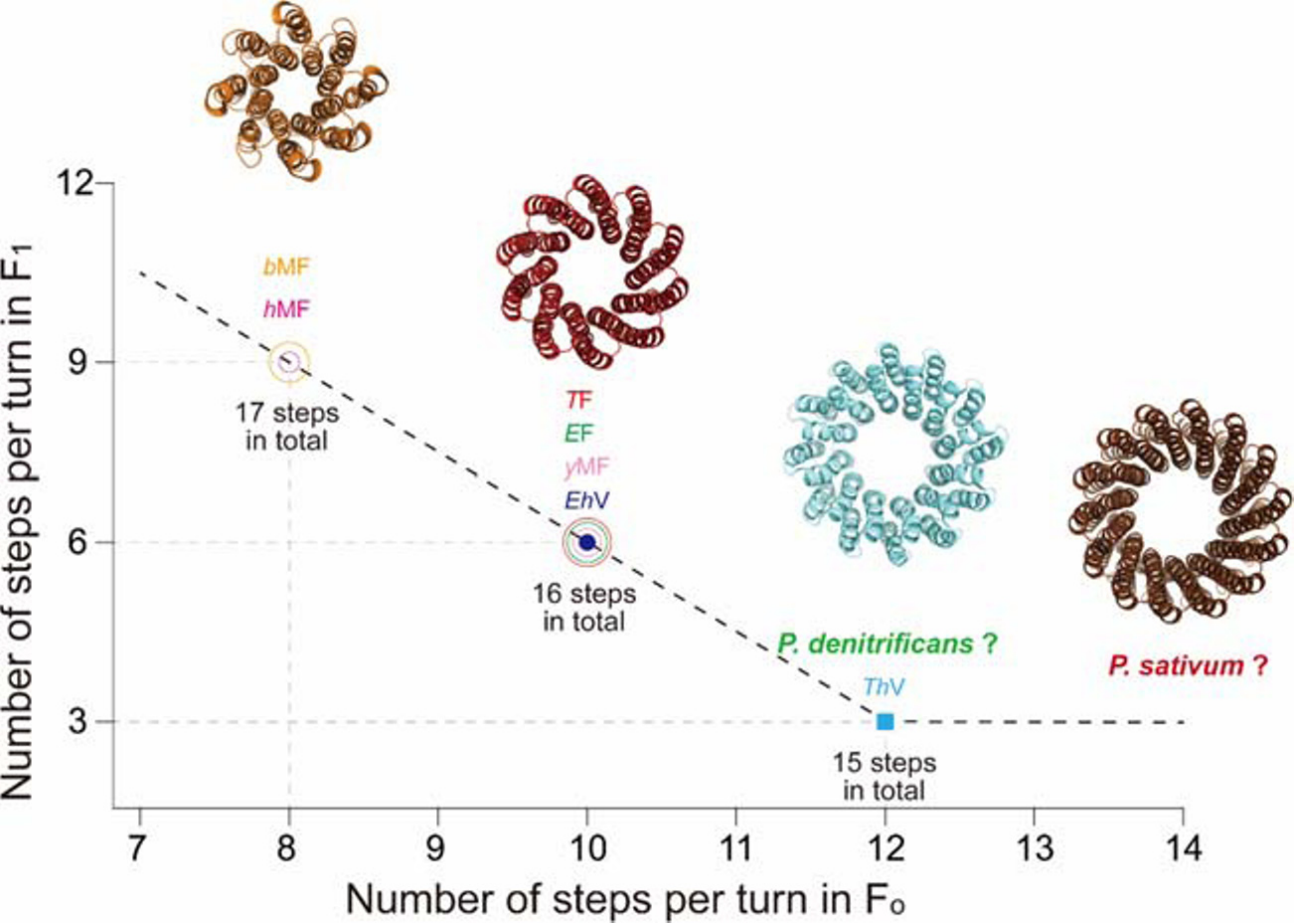
The number of steps in F_1_ versus the number of steps in F_o_. *T*F represents data on ATP synthase from thermophilic *Bacillus* PS3, *E*F from *Escherichia coli, y*MF from yeast, *b*MF from bovine, *h*MF from human, *Eh*V from *Enterococcus hirae*, and *Th*V from *Thermus thermophilus*. Structures of *c*_8_-ring of *b*MF (orange), *c*_10_-ring of *T*F (red), *c*_12_-ring of *Th*V (cyan), and *c*_14_-ring of *Pisum sativum* ATP synthase (brown) are shown

We analyzed the data on the following ATP synthases: thermophilic *Bacillus* PS3 *T*F, *Escherichia coli E*F, yeast *y*MF, and bovine *b*MF. We chose these ATP synthases because both single-molecule rotation assays on F_1_ (Watanabe et al. 2010; Bilyard et al. 2012; Steel et al. 2015; Kobayashi et al. 2020) and the structural data on the *c*-ring of F_o_ (Stock et al. 1999; Ballhausen et al. 2009; Watt et al. 2010; Guo et al. 2019) are available. Considering the evolutionary distance and the high-sequence homology of the *c*-subunits, it is highly likely that the ATP synthase from human mitochondria (*h*MF) also contains 8 *c*-subunits. Therefore, we added the data on *h*MF. The correlation between the number of steps in F_1_ and F_o_ is shown in Fig. 2. Clearly, a 6-step F_1_ is always paired with a 10-step F_o_, whereas a 9-step F_1_ is paired with an 8-step F_o_.

To gain more data points, we added information gained from the studies on V-ATPases, which are evolutionarily highly related rotary ATPases. V-ATPases are also composed of two distinctive domains, V_1_ and V_o_, corresponding to F_1_ and F_o_, respectively. To date, there are only two well-characterized V-ATPases for which the number of rotational steps in V_1_ and number of proton-carrying units in V_o_ is known. One of them is the *Enterococcus hirae* V-ATPase (*Eh*V), with a 6-step V_1_ (Iida et al. 2019) and a V_o_ with 10 proton-carrying units (Murata et al. 2005), providing support for the abovementioned correlation. The other one is the V-ATPase from *Thermus thermophilus* (*Th*V), which consists of a 3-step V_1_ (Furuike et al. 2011) and a V_o_ with 12 proton-carrying units (Toei et al. 2007). This data point from *Th*V appears to expand the correlation map to include 3-step ATPases paired with 12-step proton-conducting domains.

## Implications and perspective

Figure 2 shows an obvious trend: ATPase motors with more steps have proton-conducting motors with less steps. Although the total number of steps varies from 15 to 17, this trend appears to be relevant in the design principle of rotary ATPases. One possibility is that rotary ATPases are designed to have potential minima around 16. It is highly likely that some angular pause positions in F_1_/V_1_ overlap with the pause positions in F_o_/V_o_. In that case, the above numbers should indicate the maximum numbers of rotary potential minima per turn in the ATPase complex.

In this letter, we only consider the data points of F/V-ATPases, of which the number of the proteolipid is 8, 10, or 12, due to the limited information. On the other hand, some ATPase’s have different numbers of proteolipids: 9, 11, 13, 14, or 15 (Meier et al. 2005; Pogoryelov et al. 2009; Saroussi et al. 2012; Preiss et al. 2014, 2015). Therefore, it is important to analyze other ATPases to investigate the universality and limitation of the found correlation between the step numbers of F_1_ and F_o_. At least, the correlation line in Fig. 2 should be kinked or broken for F_o_/V_o_ with proteolipids more than 12, because the number of rotational steps in F_1_/V_1_ should not be 2 or less, considering the conservation of the threefold symmetry of F_1_ without exception. A simple expectation is that when the number of proteolipids is 12 or more, F_1_/V_1_ is a 3-step motor.

In this regard, F_o_F_1_ from *Caldalkalibacillus thermarum* TA2.A1 (*Ct*F) could be along this contention: *Ct*F_o_ has 13 proteolipids in the c-ring (Matthies et al. 2009), and the single-molecule rotation assay of *Ct*F_1_ found only 3 distinctive pauses per turn. It should be mentioned that a few rotation trajectories of *Ct*F_1_ seem to show a sign of the additional pauses in a turn. A more conclusive analysis is awaited. It would be also interesting to characterize the rotary catalysis of the F-ATPases from *Paracoccus denitrificans* (Morales-Rios et al. 2015)*, Pisum sativum* (chloroplast) (Saroussi et al. 2012), and cyanobacteria bacteria species (Pogoryelov et al. 2007), in which the F_o_ contains 12, 14, and 13–15 *c-*subunits, respectively. It should be noted that the deviance from the found correlation may come from ATPases isolated from cyanobacteria species: the single-molecule rotation assay on a thermophilic cyanobacteria species shows the ADP-inhibition pause at a difference position from ATP-binding pause found in active rotation (Konno et al. 2006), suggesting cyanobacterial F_1_ make more than 3 steps per turn.

## Summary

Recent progress in single-molecule rotation analysis and structural analysis on rotary ATPases has revealed a variety of functions and structures among species. This allows for comprehensive analyses. Here, we report a correlation between the number of steps in F_1_/V_1_ and that in F_o_/V_o_. There is a clear trend showing that ATPase motors with more steps have proton-conducting motors with less steps. In addition, ATPases with 6 steps are always paired with proton-conducting domains with 10 steps. To confirm the universality of these findings, we need more data on the rotation and structure of rotary ATPases. A theoretical approach is also needed to investigate the mechanism behind these rules.
